# Application of RNA-Seq transcriptome analysis: CD151 is an Invasion/Migration target in all stages of epithelial ovarian cancer

**DOI:** 10.1186/1757-2215-5-4

**Published:** 2012-01-24

**Authors:** Rebecca A Mosig, Li Lin, Emir Senturk, Hardik Shah, Fei Huang, Peter Schlosshauer, Samantha Cohen, Robert Fruscio, Sergio Marchini, Maurizio D'Incalci, Ravi Sachidanandam, Peter Dottino, John A Martignetti

**Affiliations:** 1 Department of Genetics and Genomic Sciences, Mount Sinai School of Medicine, New York, NY, USA; 2 Department of Pathology, Mount Sinai School of Medicine, New York, NY, USA; 3 Division of Gynecologic Oncology, Mount Sinai School of Medicine, New York, NY, USA; 4 San Gerardo Hospital, University of Milano-Bicocca, Monza, Italy; 5 Department of Oncology, Instituto "Mario Negri", Milano, Italy; 6 Mario Negri Gynecological Oncology Group (MaNGO), Milano, Italy

**Keywords:** CD151, Epithelial Ovarian Cancer, Invasion, Migration, Metastasis, RNA-Seq

## Abstract

**Background:**

RNA-Seq allows a theoretically unbiased analysis of both genome-wide transcription levels and mutation status of a tumor. Using this technique we sought to identify novel candidate therapeutic targets expressed in epithelial ovarian cancer (EOC).

**Methods:**

Specifically, we sought candidate invasion/migration targets based on expression levels across all tumors, novelty of expression in EOC, and known function. RNA-Seq analysis revealed the high expression of CD151, a transmembrane protein, across all stages of EOC. Expression was confirmed at both the mRNA and protein levels using RT-PCR and immunohistochemical staining, respectively.

**Results:**

In both EOC tumors and normal ovarian surface epithelial cells we demonstrated CD151 to be localized to the membrane and cell-cell junctions in patient-derived and established EOC cell lines. We next evaluated its role in EOC dissemination using two ovarian cancer-derived cell lines with differential levels of CD151 expression. Targeted antibody-mediated and siRNA inhibition or loss of CD151 in SKOV3 and OVCAR5 cell lines effectively inhibited their migration and invasion.

**Conclusion:**

Taken together, these findings provide the first proof-of-principle demonstration for a next generation sequencing approach to identifying candidate therapeutic targets and reveal CD151 to play a role in EOC dissemination.

## Background

Epithelial ovarian cancer (EOC) is the most common cause of gynecologic cancer death and the fifth most lethal cancer among women [1]. Despite a relatively low occurrence rate (1 in 72) compared to other female cancers, the low 5-year survival rate of ~40% translates to greater than 14,000 yearly deaths from ovarian cancer in the United States [1]. One main contributor to the low survival rate is the late stage at which EOC is usually detected: upwards of 80% of EOC is discovered after localized spread. When detected early, the EOC 5-year survival rate is ~90% [2].

Beyond earlier diagnosis and detection, the identification of novel therapeutic targets or approaches to overcome chemoresistance is necessary to treat late stage or recurrent disease that will occur even with more sensitive and specific screening and detection methods. Since the introduction of platinum-based drugs as first line chemotherapy in the early 1980's followed by the addition of taxane containing agents in the mid-1990's, there has been little change to the first line treatment of EOC [3]. Novel administration methods, such as intraperitoneal therapy, and dosing, such as dose-dense taxol, have yielded slight improvements in progression-free survival and overall survival [3]. Molecularly targeted therapies to treat recurrent and/or chemoresistant disease show some promise but large conclusive trials have not been completed [3]. Therefore, the need for new targets and drugs remains high [1].

Next Generation Sequencing technology is now allowing for the thorough and unbiased profiling of a number of cancer genomes and transcriptomes [4-7]. Analysis of mutational profiles, copy number variations, and expression profiles have yielded insights into commonly affected genes and pathways important for carcinogenesis in a number of cancers including melanoma, pancreatic, lung, and breast cancers [4-7]. Theoretically, by applying RNA-Seq technology to ovarian cancers relevant pathways and molecules for therapeutic intervention should be identifiable.

CD151 is an integral membrane protein and member of the tetraspanin family. It associates with integrins and other transmembrane proteins tetraspanin-enriched microdomains, thereby playing a role in cell-matrix or cell- cell attachment [8,9]. Migration signaling pathways including PI3K, FAK, and Rho/Src mediate cell behavior in response to CD151 interactions [10-18]. Functionally, a CD151-targeting antibody was shown to inhibit cell migration in an *in vivo *breast cancer xenograft model and contain breast cancer cells within a single contiguous tumor [19].

Using a highly clinically annotated sample set of EOC, representing both early and late stage tumors, we interrogated global expression patterns by RNA-Seq. We identified the transmembrane protein CD151 as being overexpressed in all tumor samples and then demonstrated it to be a potential target to inhibit metastasis and dissemination of ovarian cancer.

## Methods

### Patients and Specimen Collection

EOC tumor samples and ascites cells were collected from MSSM and San Gerardo Hospital patients at the time of surgery under their respective IRB-approved protocols, as previously described [20]. Samples were divided in the operating room and a portion sent for pathology confirmation and staging. A portion was flash frozen for subsequent RNA and protein analysis, and another portion used for generating patient-derived cell lines. For the RNA-Seq "discovery set", **16 papillary serous tumor samples representative of all stages of the disease (3 stage I/II, 8 stage III, 1 stage IV, 2 peritoneal metastatic lesions, and 2 recurrent tumors) and two borderline serous tumors were collected and analyzed**. An additional set of 25 tumors (6 stage I/II, 7 stage III/IV, 8 peritoneal metastatic lesions, and 4 recurrent tumors) were used as a "validation set".

### RNA extraction

RNA was extracted from frozen tissue using QIAzol according to manufacturer's instructions (Qiagen, Valencia, California). Briefly, tissue was homogenized in QIAzol on ice. Chloroform was added, mixed and centrifuged to allow for separation and removal of the aqueous layer. RNA was precipitated in isopropanol overnight at -20°C. The suspension was centrifuged to pellet the RNA, washed with 75% ethanol and then resuspended in RNAase-free water. RNA integrity numbers (RINs) were analyzed using the Agilent Bioanalyzer and only RNA with a RIN of > 8.0 was submitted for next-generation sequencing.

### RNA-Seq

Epithelial ovarian cancer transcriptomes were prepared for paired-end sequencing on the Illumina GAII platform using the manufacturer's protocols and with a second size selection step to reduce ligation artifacts. Reads were aligned using Eland32 (provided with the Illumina sequencing platform). Expression levels were quantified by running ERANGE v. 3.0.2. [21]. For each gene, ERANGE reported the number of mapped reads per kilobase of exon per million mapped reads (RPKM).

### Quantitative Real-time Reverse Transcription PCR

RNA-Seq data was confirmed by quantitative real-time PCR. One microgram of RNA was reverse transcribed to cDNA using the BioRad Iscript system (Biorad, Hercules, California). Quantitative real-time PCR was performed on an ABI PRISM 7900 HT sequence detection system (Applied Biosystems, Carlsbad, California). Cycle number values were normalized against two housekeeping genes, B2 M and GAPDH. Data shown is the average of three separate experiments, each performed in triplicate. The CD151 primers used were CD151 Fwd: 5'- AGACAGCTGCTGCAAGAC-3' and CD151 Rev: 5'-TGGATGAAGGTCTCCAACT-3'.

### Immunohistochemistry and Fluorescent Immunocytochemistry

Four-micrometer thick tumor sections were stained with α-CD151 antibody (Cat # NCL-CD151, Leica, Wetzlar, Germany) and the R&D mouse cell and tissue DAB staining kit and counterstained with Hematoxylin. A murine IgG1 isotype control antibody (Clone 11711, R&D systems MAB002) was used as a negative experimental control.

CD151 expression levels and subcellular localization were examined in patient-derived and commercially available tumor cell lines using the α-CD151 antibody (Cat #NCL-CD151, Leica, Wetzlar, Germany). Alexa fluor- 548 goat anti-mouse secondary (Cat #A21137, Invitrogen, Carlsbad, California) was used and nuclear counterstaining was performed with Vectashield mounting medium for fluorescence with Dapi (Cat #H1200, Vector Laboratories, Burlingame, California).

### Generation of Low Passage Number Ascites Cell Lines and Cell Culture

Ascites fluid was centrifuged for 10 minutes at low speed at 4°C to pellet the cellular fraction. Cells were resuspended in DMEM containing 10% FBS and Penicillin-Streptomycin and allowed to adhere. Media was changed daily until cells reached confluence at which time they were passaged. RNA and immunostaining procedures were performed using only 2nd or 3rd passage cells.

### SiRNA Transfection

SMARTPool siRNA targeting human CD151 (Dharmacon, Lafayette, Colorado) was transfected into SKOV3 or OVCAR5 cells using Lipofectamine as described previously [22]. Knockdown was confirmed at the RNA level using qRT-PCR and at the protein level using IHC as described above.

### Migration and Invasion Assays

Migration and invasion experiments were performed according to the manufacturer's recommended protocol (BD Biosciences, Franklin Lakes, New Jersey). Briefly, SKOV3 or OVCAR5 cells were resuspended in serum free media with or without mouse α-CD151 or control mouse α-V5 antibody (Invitrogen, Carlsbad, California) in the upper chamber of modified Boyden chamber transwells. Bottom chambers were filled with media containing 10% FBS as a chemoattractant. Cells were allowed to migrate or invade through matrigel for 24 hours followed by calcein dye staining and visualization using a fluorescent detector. Results shown are the averages of 3 separate experiments performed in triplicate. Statistical significance was measured using the Student's T test with p < 0.05 considered significant.

## Results

### CD151 is expressed across all stages of EOC

A total of **16 papillary serous **epithelial ovarian tumors, representing early- and late-stage disease and metastatic nodules and recurrences, **and two borderline ovarian tumors **were selected for RNA-Seq in our "discovery set" of samples. Data for ~10,000 transcripts with an average expression coverage level greater than one across all samples was achieved. Coverage is a representation of the number of sequence reads mapping to the exonic regions of a gene adjusted for the overall transcript length. Our search for candidates was defined by: 1) representation in all samples, 2) high transcript abundance (top 5%), 3) lack of previous identification in EOC, and 4) potential functionality as a treatment target. Based on these search criteria, CD151 became a high-ranking candidate including the fact that it ranked as high as the top 2% of genes expressed in our samples (Table 1). The ability of CD151 to affect cell dissociation and migration in other tumor models and our novel discovery of its expression in EOC made CD151 a good proof-of-principle candidate for further study.

**Table 1:** RNA-Seq tumor coverage values

Disease Group	Mean	Range	N#
Borderline	13.993	8.170-19.815	2
Early EOC	34.820	22.525-48.960	3
Late EOC	24.888	14.280-51.035	9
Metastatic	15.375	10.740-20.010	2
Recurrent	21.418	14.340-28.495	2

To confirm these RNA-Seq results, we analyzed CD151 expression using quantitative real-time RT-PCR (qRT-PCR) in each of the discovery set of tumors that had been sequenced (Table 2). In addition to these 18 samples, 25 additional **papillary serous **tumors, not used for RNA-Seq, were then analyzed as a "validation set". While RNA-Seq expression levels and the qRT-PCR-measured expression levels did not always correlate precisely, all tumors expressed CD151 in readily detectable amounts. One explanation for the variation may be that the real-time values are normalized to B2M and GAPDH, whose expression may vary between tumor samples [23]. In fact, in our RNA- Seq sample set, the B2M and GAPDH transcript levels also varied markedly. B2M coverage levels ranged greater than 30 fold between samples and GAPDH levels ranged greater than 10 fold (data not shown).

**Table 2:** qRT-PCR tumor expression values

	-----------------Discovery-----------------			----------------Validation----------------		
**Disease Group**	**Mean**	**Range**	**N#**	**Mean**	**Range**	**N#**

Borderline	0.038	0.019-0.056	2	-	-	-
Early EOC	0.257	0.046-0.495	3	0.050	0.014-0.090	6
Late EOC	0.404	0.003-2.45	9	1.022	0.085-3.410	7
Metastatic	0.708	0.497-0.920	2	1.160	0.039-1.231	8
Recurrent	0.122	0.005-0.239	2	0.365	0.031-3.605	4

Overall, CD151 was expressed across all stages and no differences were noted with either increasing stage or a particular subtype (Table 1). One trend of note, given the relatively small sample set in this study, was that primary tumors, regardless of stage, on average expressed higher levels of CD151 than either recurrent tumors or metastatic lesions presenting at time of primary debulking surgery (27.4 v 18.4, respectively, p = 0.11). Borderline tumors possessed the lowest average coverage values (14.0).

### CD151 is expressed in EOC tumors and normal ovary surface epithelial cells

**Figure 1:**
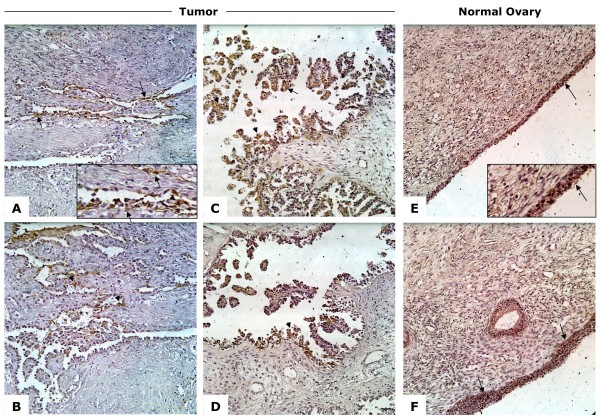
**CD151 is expressed in EOC tumors and normal ovary surface epithelial cells**. a-d) Membranous (arrows) and cytoplasmic (arrowheads) CD151 staining in early stage epithelial ovarian cancer. e-f) Primarily membranous (arrows) staining in the ovarian surface epithelial cell layer of a normal ovary. Insets in a) and e) show higher magnification and staining detail. Insets show higher maginifcation images.

Having identified overexpression on the RNA level, we next evaluated the expression of CD151 protein in tumors and normal ovary tissues using immunohistochemistry. Previous reports revealed that CD151 localizes to either the cell membrane or within the cytoplasm of cells in a context specific manner [9,24-26]. Similar to both breast and colorectal cancers [10,12,27-29], CD151 staining in ovarian tumors was seen to be both membranous and cytoplasmic (Figure 1). CD151 was also present in normal ovarian surface epithelial cells where it was primarily expressed on the membrane (Figure 1).

### CD151 is expressed in ascites-derived and EOC cell lines and immortalized ovarian surface epithelial cells (IOSE) and is localized to cell-cell junctions

**Figure 2:**
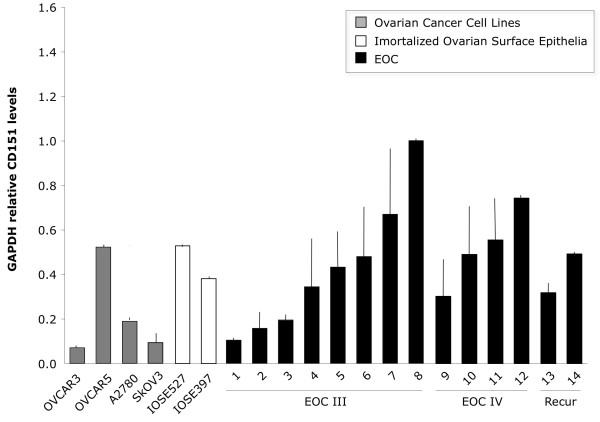
**CD151 qRT-PCR mRNA expression levels in patient-derived ascites cell lines**. Patient-derived ascites cell lines, immortalized OSE, and EOC cell lines express CD151.

The known role of CD151 in cell-cell attachment and its potential role in invasion/migration in ovarian cancer led us next to examine the expression of CD151 in ovarian-derived cell lines. These included ascites-derived lines that we had established from patients with ovarian cancer, commercially available EOC cell lines (A2780, OVCAR3, OVCAR5 and SKOV3), and a number of immortalized ovarian surface epithelial cell lines (IOSE, IOSE397 and IOSE527). Quantitative RT-PCR revealed that all ascites-derived cell lines and ovarian cancer cells expressed CD151 message (Figure 2). In accord with our finding that ovarian surface epithelial cells express CD151 (Figure 1), IOSE also express CD151. The established ovarian cell lines expressed similar levels of CD151 compared to the primary ascites-derived lines and the immortalized OSE lines (Figure 2).

**Figure 3:**
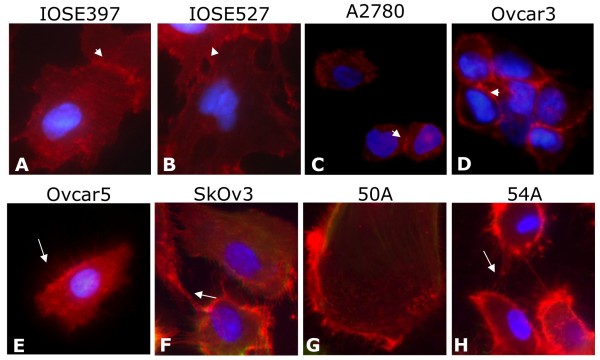
**Ascites cells and EOC cell lines express CD151 *in vitro***. CD151 localizes to the cell membrane with specificlocalization to cell-cell junctions (white arrowheads) and cell membrane extensions (white arrows) in IOSE397 (a), IOSE527 (b), A2780 (c), OVCAR3 (d), OVCAR5 (e), and SKOV3 (f) and patient derived cells 50A (g) and 54A (h). Original magnification 60X.

Immunocytochemical fluorescent staining of primary ovarian ascites cell lines and EOC and IOSE cell lines revealed that all patient-derived cell lines expressed CD151 at the protein level (Figure 3). Furthermore, CD151 localized not only to the cell membrane but intriguingly was also expressed at very high concentrations at cell-cell attachment points and cell membrane extensions between cells (Figure 3). We are unaware of this association being previously described in EOC derived cell lines, although it has been reported previously in breast and epidermal carcinoma cell lines [12,16,30,31]. Nonetheless, this is in agreement with the postulated role of CD151 in mediating cell attachment and migration.

### Migration and invasion of ovarian cancer cell lines is blocked by inhibition of CD151

**Figure 4:**
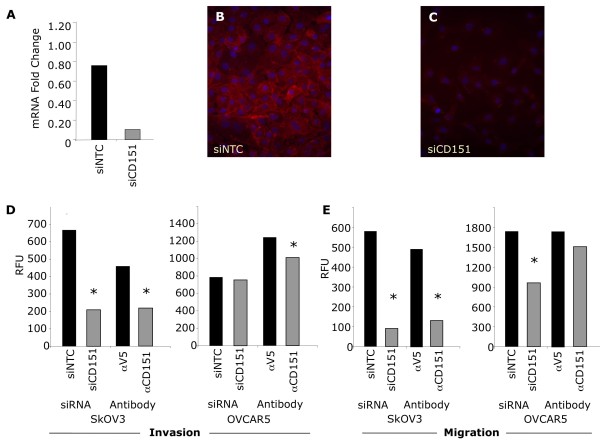
**Silencing and/or blocking of CD151 impedes SKOV3 and OVCAR5 migration and invasion**. A-C) CD151 knockdown with siCD151 transfection at the mRNA (a) and protein (b-c) levels. d) Reduction of SKOV3 and OVCAR5 migration through transwell membranes in response to either siCD151 transfection or αCD151 antibody treatment. e) Reduction of SKOV3 and OVCAR5 invasion through matrigel and transwell membranes in response to either siCD151 transfection or treatment with αCD151 antibody. *p < 0.01. Results are representative of 3 separate experiments performed in triplicate.

The postulated role of CD151 in cell migration and tumor spread led us to test if blocking CD151 expression or function could inhibit ovarian cell migration and invasion. Therefore, for these experiments we used either siRNA- mediated knockdown or a specific CD151 antibody. We chose the SKOV3 (lower expression) and OVCAR5 (higher expression) cell lines for their differential RNA expression of CD151 (Figure 2). Using a pool of siRNA oligonucleotides targeting CD151, titration experiments revealed optimal knockdown of CD151 mRNA (90% knockdown) and protein occurred 96 hours after transfection (Figure 4a-c). Both invasion and migration of siCD151- transfected SKOV3 cells over 24 hours were significantly reduced following inhibition of CD151 (Figure 4d-e). We also tested the inhibition of invasion and migration in the higher expressing OVCAR5 cell line. While siRNA- mediated inhibition of CD151 in OVCAR5 cells significantly reduced their migration, we found no evidence of an effect on invasion (Figure 4d-e).

A second method of CD151 inhibition, antibody-mediated, also significantly inhibited the ability of SKOV3 cells to invade and migrate (Figure 4d-e). In the CD151 higher expressing OVCAR5 cells, antibody-mediated blockade, at the concentrations used, had only an ~ 20% decrease in invasion but no measurable effect on migration (Figure 4d-e).

## Discussion

These studies are the first to demonstrate that the tetraspanin CD151 is expressed in epithelial ovarian cancers and that its expression is independent of stage or histological subtype. We analyzed EOC tumor expression levels of all RNA transcripts in an unbiased manner through the use of RNA-Seq with the goal of identifying possible novel therapeutic targets. From this analysis of 18 whole transcriptomes we identified high CD151 expression across all stages and subtypes of EOC, including borderline ovarian tumors. We then confirmed expression of CD151 in this discovery set and a second set of tumor tissues using qRT-PCR and immunohistochemistry. The previously described role of CD151 as a possible anti-metastatic target led us to further examine the role of CD151 in ovarian cancer and its migration and invasion in culture.

Immunohistochemical staining of EOC tumors and normal ovary showed CD151 expression in tumor cells as well as normal OSE cells. Immunocytochemistry of cells from patient ascites fluid showed CD151 to be localized to the cell periphery and highly concentrated at cell-cell junction points and along outstretched cellular elongations. Finally, functional studies showed that SKOV3 and OVCAR5 cell invasion and migration are differentially inhibited by α-CD151 antibody or siRNA-mediated knockdown of CD151. It appears from these experiments that the level of CD151 expression may affect our ability to inhibit invasion and migration and therefore CD151 blockade may need to be titrated to achieve the most robust inhibition.

CD151 has previously been postulated to play important roles in a number of cancers including colorectal and breast cancers but no studies had previously identified or examined its expression and function in ovarian cancer. In colorectal cancer CD151 is differentially expressed between normal (high expression), primary (low expression), and metastatic (high expression) tissue [28]. It is proposed that these changes are part of a hypoxic response that drives cell detachment and migration [28]. In our own discovery sample set of EOC tumors, while not reaching statistical significance, we did find an interesting trend of higher CD151 expression in primary ovarian tumors and relatively lower expression in disseminated metastatic or recurrent disease (Tables 1 and 2). It is possible that this is due to different environmental effects in secondary sites, such as hypoxia or surrounding cell types. This hypothesis will need to be expanded upon by examination of a larger collection of samples.

The expression of tetraspanin CD151 on the cell surface of EOC cells may play a role in the spread of these cells to other organs in the peritoneal cavity. In xenograft tumor models, antibody-mediated inhibition of CD151 has been shown to hinder the spread of metastatic tumors, reinforcing the idea that CD151 is functionally important for either cell detachment from a tumor or migration away from that tumor [19,32]. Intriguingly, in breast cancer models comparing CD151-expressing cells against CD151-ablated cells, tumor growth was delayed in the absense of CD151 [12]. In immunohistochemical studies of breast cancer, staining varied greatly in both intensity and cellular localization, which may reflect the heterogeneity of the tumor cells' environment or activity [33]. CD151 staining intensity and localization in normal OSE and ovarian tumor cells also varies. CD151 protein was localized to both the membrane and the cytoplasm of cancer cells (Figure 1). In contrast, in normal ovarian surface CD151 localized mostly to the cell membrane. It is possible that the internalization of CD151 into cytoplasmic endosomes may reduce its ability to cooperate with other binding partners and cells, allowing detachment from the primary tumor [25]. On ascites-derived patient cell lines, IOSE, and EOC cell lines, CD151 not only localized to the membrane but more specifically to the cell-cell junctions and along cell membrane extensions (Figure 3). Although *in vitro *cellular localization suggests no difference between cancerous cell lines and OSE cells, this may due to distinct conditions in cell culture such as attachment to plastic and tightly controlled O2/CO2 balance.

Expression of CD151 in EOC tumors, the known involvement of CD151 in cell migration and invasion, and the *in vivo *ability of αCD151 antibody to contain breast cancer tumors to single nodules and eliminate tumor spread suggested that CD151 may also represent a promising and relevant candidate for therapeutic targeting in ovarian cancer. We show that in the ovarian cancer cell lines SKOV3 and OVCAR5, CD151 is a functionally important molecule whose silencing or blockade impeded cell migration or invasion to different levels dependent on level of CD151 expression. A role for CD151 in cell migration and invasion is consistent through many cancer types including breast, prostate, colorectal, and pancreatic cancers [15,19,32,34-36]. Ultimately, the value of CD151 as a therapeutic target in EOC will need to be demonstrated in *in vivo *studies.

## Abbreviations

EOC: epithelial ovarian cancer; RIN: RNA integrity number; IOSE: immortalized ovarian surface epithelial.

## Competing interests

The authors declare that they have no competing interests.

## Authors' contributions

RAM participated in sample collection, selection, sequencing analysis, and all molecular studies and manuscript drafting. LL participated in all molecular studies. ES participated in sample collection and sequencing analysis. HS provided bioinformatics support and analysis. FH participated in sample collection and cell-line derivation. PS provided pathological staging and staining analysis. SC supplied samples and clinical information. RF, SM, and MD supplied samples and clinical information and participated in study design. RS provided bioinformatics support and analysis as well as data interpretation. PD supplied samples and clinical information and participated in study design. JAM participated in overall study design, sample selection, sequencing analysis, and preparation of the manuscript. All Authors reviewed and approved the final version of the manuscript.

## References

[B1] Altekruse SF Kosary CL Krapcho M Neyman N Aminou R Waldron W Ruhl J Howlader N Tatalovich Z Cho H Mariotto A Eisner MP Lewis DR Cronin K Chen HS Feuer EJ Stinchcomb DG Edwards BK SEER Cancer Statistics Review, 1975-2007 [Internet] Bethesda, MD: National Cancer Institute

[B2] Cannistra SA Cancer of the ovary N Engl J Med 2004 351 2519 29 10.1056/NEJMra041842 15590954

[B3] Guarneri V Piacentini F Barbieri E Conte PF Achievements and unmet needs in the management of advanced ovarian cancer Gynecol Oncol 2010 117 152 8 10.1016/j.ygyno.2009.11.033 20056266

[B4] Pleasance ED Stephens PJ O'Meara S McBride DJ Meynert A Jones D Lin ML Beare D Lau KW Greenman C Varela I Nik-Zainal S Davies HR Ordonez GR Mudie LJ Latimer C Edkins S Stebbings L Chen L Jia M Leroy C Marshall J Menzies A Butler A Teague JW Mangion J Sun YA McLaughlin SF Peckham HE Tsung EF Costa GL Lee CC Minna JD Gazdar A Birney E Rhodes MD McKernan KJ Stratton MR Futreal PA Campbell PJ A small-cell lung cancer genome with complex signatures of tobacco exposure Nature 2010 463 184 90 10.1038/nature08629 20016488 PMC2880489

[B5] Timmermann B Kerick M Roehr C Fischer A Isau M Boerno ST Wunderlich A Barmeyer C Seemann P Koenig J Lappe M Kuss AW Garshasbi M Bertram L Trappe K Werber M Herrmann BG Zatloukal K Lehrach H Schweiger MR Somatic mutation profiles of MSI and MSS colorectal cancer identified by whole exome next generation sequencing and bioinformatics analysis PLoS One 2010 5 e15661 10.1371/journal.pone.0015661 21203531 PMC3008745

[B6] Pleasance ED Cheetham RK Stephens PJ McBride DJ Humphray SJ Greenman CD Varela I Lin ML Ordonez GR Bignell GR Ye K Alipaz J Bauer MJ Beare D Butler A Carter RJ Chen L Cox AJ Edkins S Kokko-Gonzales PI Gormley NA Grocock RJ Haudenschild CD Hims MM James T Jia M Kingsbury Z Leroy C Marshall J Menzies A Mudie LJ Ning Z Royce T Schulz-Trieglaff OB Spiridou A Stebbings LA Szajkowski L Teague J Williamson D Chin L Ross MT Campbell PJ Bentley DR Futreal PA Stratton MR A comprehensive catalogue of somatic mutations from a human cancer genome Nature 2010 463 191 6 10.1038/nature08658 20016485 PMC3145108

[B7] Bignell GR Greenman CD Davies H Butler AP Edkins S Andrews JM Buck G Chen L Beare D Latimer C Widaa S Hinton J Fahey C Fu B Swamy S Dalgliesh GL Teh BT Deloukas P Yang F Campbell PJ Futreal PA Stratton MR Signatures of mutation and selection in the cancer genome Nature 2010 463 893 8 10.1038/nature08768 20164919 PMC3145113

[B8] Hasegawa M Furuya M Kasuya Y Nishiyama M Sugiura T Nikaido T Momota Y Ichinose M Kimura S CD151 dynamics in carcinoma-stroma interaction: Integrin expression, adhesion strength and proteolytic activity Lab Invest 2007 87 882 92 10.1038/labinvest.3700657 17632541

[B9] Liu L He B Liu WM Zhou D Cox JV Zhang XA Tetraspanin CD151 promotes cell migration by regulating integrin trafficking J Biol Chem 2007 282 31631 42 10.1074/jbc.M701165200 17716972

[B10] Yang XH Flores LM Li Q Zhou P Xu F Krop IE Hemler ME Disruption of laminin-integrin-CD151-focal adhesion kinase axis sensitizes breast cancer cells to ErbB2 antagonists Cancer Res 2010 70 2256 63 10.1158/0008-5472.CAN-09-4032 20197472 PMC3310185

[B11] Zuo H Liu Z Liu X Yang J Liu T Wen S Zhang XA Cianflone K Wang D CD151 gene delivery after myocardial infarction promotes functional neovascularization and activates FAK signaling Mol Med 2009 15 307 15 19603100 10.2119/molmed.2009.00025 PMC2710288

[B12] Yang XH Richardson AL Torres-Arzayus MI Zhou P Sharma C Kazarov AR Andzelm MM Strominger JL Brown M Hemler ME CD151 accelerates breast cancer by regulating alpha 6 integrin function, signaling, and molecular organization Cancer Res 2008 68 3204 13 10.1158/0008-5472.CAN-07-2949 18451146 PMC4764302

[B13] Sawada S Yoshimoto M Odintsova E Hotchin NA Berditchevski F The tetraspanin CD151 functions as a negative regulator in the adhesion-dependent activation of ras J Biol Chem 2003 278 26323 6 10.1074/jbc.C300210200 12782641

[B14] Franco M Muratori C Corso S Tenaglia E Bertotti A Capparuccia L Trusolino L Comoglio PM Tamagnone L The tetraspanin CD151 is required for met-dependent signaling and tumor cell growth J Biol Chem 2010 10.1074/jbc.M110.145417 PMC2998140 20937830

[B15] Zhu GH Huang C Qiu ZJ Liu J Zhang ZH Zhao N Feng ZZ Lv XH Expression and prognostic significance of CD151, c-met, and integrin alpha3/alpha6 in pancreatic ductal adenocarcinoma Dig Dis Sci 2010 10.1007/s10620-010-1416-x 20927591

[B16] Johnson JL Winterwood N DeMali KA Stipp CS Tetraspanin CD151 regulates RhoA activation and the dynamic stability of carcinoma cell-cell contacts J Cell Sci 2009 122 2263 73 10.1242/jcs.045997 19509057

[B17] Ke AW Shi GM Zhou J Wu FZ Ding ZB Hu MY Xu Y Song ZJ Wang ZJ Wu JC Bai DS Li JC Liu KD Fan J Role of overexpression of CD151 and/or c-met in predicting prognosis of hepatocellular carcinoma Hepatology 2009 49 491 503 10.1002/hep.22639 19065669

[B18] Yamada M Sumida Y Fujibayashi A Fukaguchi K Sanzen N Nishiuchi R Sekiguchi K The tetraspanin CD151 regulates cell morphology and intracellular signaling on laminin-511 FEBS J 2008 275 3335 51 10.1111/j.1742-4658.2008.06481.x 18492066

[B19] Zijlstra A Lewis J Degryse B Stuhlmann H Quigley JP The inhibition of tumor cell intravasation and subsequent metastasis via regulation of in vivo tumor cell motility by the tetraspanin CD151 Cancer Cell 2008 13 221 34 10.1016/j.ccr.2008.01.031 18328426 PMC3068919

[B20] Marchini S Mariani P Chiorino G Marrazzo E Bonomi R Fruscio R Clivio L Garbi A Torri V Cinquini M Dell'Anna T Apolone G Broggini M D'Incalci M Analysis of gene expression in early-stage ovarian cancer Clin Cancer Res 2008 14 7850 60 10.1158/1078-0432.CCR-08-0523 19047114

[B21] Mortazavi A Williams BA McCue K Schaeffer L Wold B Mapping and quantifying mammalian transcriptomes by RNA-seq Nat Methods 2008 5 621 8 10.1038/nmeth.1226 18516045 PMC13303166

[B22] Mosig RA Dowling O Difeo A Ramirez MC Parker IC Abe E Diouri J Aqeel AA Wylie JD Oblander SA Madri J Bianco P Apte SS Zaidi M Doty SB Majeska RJ Schaffler MB Martignetti JA Loss of MMP-2 disrupts skeletal and craniofacial development and results in decreased bone mineralization, joint erosion and defects in osteoblast and osteoclast growth Hum Mol Genet 2007 16 1113 23 10.1093/hmg/ddm060 17400654 PMC2576517

[B23] Tricarico C Pinzani P Bianchi S Paglierani M Distante V Pazzagli M Bustin SA Orlando C Quantitative real-time reverse transcription polymerase chain reaction: Normalization to rRNA or single housekeeping genes is inappropriate for human tissue biopsies Anal Biochem 2002 309 293 300 10.1016/S0003-2697(02)00311-1 12413463

[B24] Lineberry N Su L Soares L Fathman CG The single subunit transmembrane E3 ligase gene related to anergy in lymphocytes (GRAIL) captures and then ubiquitinates transmembrane proteins across the cell membrane J Biol Chem 2008 283 28497 505 10.1074/jbc.M805092200 18713730 PMC2568916

[B25] Rana S Claas C Kretz CC Zoeller M Activation-induced internalization differs for the tetraspanins CD9 and Tspan8: Impact on tumor cell motility Int J Biochem Cell Biol 2010 10.1016/j.biocel.2010.10.002 20937409

[B26] Yang X Claas C Kraeft SK Chen LB Wang Z Kreidberg JA Hemler ME Palmitoylation of tetraspanin proteins: Modulation of CD151 lateral interactions, subcellular distribution, and integrin-dependent cell morphology Mol Biol Cell 2002 13 767 81 10.1091/mbc.01-05-0275 11907260 PMC99597

[B27] Klosek SK Nakashiro K Hara S Goda H Hasegawa H Hamakawa H CD151 regulates HGF-stimulated morphogenesis of human breast cancer cells Biochem Biophys Res Commun 2009 379 1097 100 10.1016/j.bbrc.2009.01.023 19159612

[B28] Chien CW Lin SC Lai YY Lin BW Lin SC Lee JC Tsai SJ Regulation of CD151 by hypoxia controls cell adhesion and metastasis in colorectal cancer Clin Cancer Res 2008 14 8043 51 10.1158/1078-0432.CCR-08-1651 19073968

[B29] Sauer G Kurzeder C Grundmann R Kreienberg R Zeillinger R Deissler H Expression of tetraspanin adaptor proteins below defined threshold values is associated with in vitro invasiveness of mammary carcinoma cells Oncol Rep 2003 10 405 10 12579280

[B30] Sharma C Yang XH Hemler ME DHHC2 affects palmitoylation, stability, and functions of tetraspanins CD9 and CD151 Mol Biol Cell 2008 19 3415 25 10.1091/mbc.E07-11-1164 18508921 PMC2488315

[B31] Shigeta M Sanzen N Ozawa M Gu J Hasegawa H Sekiguchi K CD151 regulates epithelial cell-cell adhesion through PKC- and Cdc42-dependent actin cytoskeletal reorganization J Cell Biol 2003 163 165 76 10.1083/jcb.200301075 14557253 PMC2173453

[B32] Testa JE Brooks PC Lin JM Quigley JP Eukaryotic expression cloning with an antimetastatic monoclonal antibody identifies a tetraspanin (PETA-3/CD151) as an effector of human tumor cell migration and metastasis Cancer Res 1999 59 3812 20 10447000

[B33] Sadej R Romanska H Baldwin G Gkirtzimanaki K Novitskaya V Filer AD Krcova Z Kusinska R Ehrmann J Buckley CD Kordek R Potemski P Eliopoulos AG Lalani e Berditchevski F CD151 regulates tumorigenesis by modulating the communication between tumor cells and endothelium Mol Cancer Res 2009 7 787 98 10.1158/1541-7786.MCR-08-0574 19531562

[B34] Gesierich S Paret C Hildebrand D Weitz J Zgraggen K Schmitz-Winnenthal FH Horejsi V Yoshie O Herlyn D Ashman LK Zoller M Colocalization of the tetraspanins, CO-029 and CD151, with integrins in human pancreatic adenocarcinoma: Impact on cell motility Clin Cancer Res 2005 11 2840 52 10.1158/1078-0432.CCR-04-1935 15837731

[B35] Funakoshi T Tachibana I Hoshida Y Kimura H Takeda Y Kijima T Nishino K Goto H Yoneda T Kumagai T Osaki T Hayashi S Aozasa K Kawase I Expression of tetraspanins in human lung cancer cells: Frequent downregulation of CD9 and its contribution to cell motility in small cell lung cancer Oncogene 2003 22 674 87 10.1038/sj.onc.1206106 12569360

[B36] Tokuhara T Hasegawa H Hattori N Ishida H Taki T Tachibana S Sasaki S Miyake M Clinical significance of CD151 gene expression in non-small cell lung cancer Clin Cancer Res 2001 7 4109 14 11751509

